# Norm values and psychometric properties of the brief symptom inventory-18 regarding individuals between the ages of 60 and 95

**DOI:** 10.1186/s12874-018-0631-6

**Published:** 2018-12-05

**Authors:** Katja Petrowski, Bjarne Schmalbach, Melanie Jagla, Gabriele Helga Franke, Elmar Brähler

**Affiliations:** 1 Medical Psychology and Medical Sociology, Department of Psychosomatic Medicine and Psychotherapy, University Medical Center of the Johannes Gutenberg University of Mainz, Mainz, Germany; 2 Department of Psychotherapy and Psychosomatic Medicine, Dresden University of Technology, Dresden, Germany; 3 Department of Psychology, University of Münster, Fliednerstraße 21, 48149 Münster, Germany; 4 Department of Rehabilitation Psychology, University of Applied Sciences Magdeburg-Stendal, Stendal, Germany; 5 Department of Medical Psychology and Medical Sociology, University of Leipzig, Leipzig, Germany; 6 Department Psychosomatic Medicine, University of Mainz, Mainz, Germany

**Keywords:** BSI-18, 60 to 95 years of age, Psychological distress, Elderly individuals, Norm values, Psychometric properties

## Abstract

**Objective:**

The SCL-90 and the SCL-90-R are the most applied measures regarding psychological distress. To reduce and prevent an overload to of the individuals, the Brief Symptom Inventory with 18 items (BSI-18) was developed based on the SCL-90. Since psychological disorders more frequently occur at an older age, there is a growing need for efficient instruments to measure distress in the elderly. However, the BSI-18’s psychometric properties, norm values, and factorial structure have not yet been investigated in this age group.

**Methods:**

The aim of this study was to evaluate the BSI-18 in a sample of elderly people and to establish norm values for this specific population. Subsequently, demographic information and BSI-18 results were collected from a sample totaling 884 (55% female, mean age of 70.75 years, *SD* = 7.08, age range = 60–95 years). The questionnaire contains three six-item scales: somatization (SOMA), anxiety (ANX), and depression (DEPR), which form a general symptom index (GSI).

**Results:**

We found an acceptable to good model fit for a three-factor-model with a general GSI factor. The BSI-18’s psychometric properties were satisfactory. Strict measurement invariance was shown for age and gender. Additionally, we found differences in psychological distress based on sociodemographic variables.

**Conclusions:**

These findings underline the growing need for preventive mechanisms for elderly people such as, e.g., (re)activating their social networks and strengthening their physical and psychological well-being.

## Introduction

Internationally, the Symptom Check List with 90 items (SCL-90) [1, 2] and its revised version (SCL-90-R) [3] are the most applied questionnaires for the assessment of psychological distress, especially in clinical practice [1]. Nevertheless, they are very long and time-consuming rating scales. As short versions of the Symptom-Checklist 90 [2], two Brief Symptom Inventory versions were developed [4, 5]. Since the SCL-90 and the SCL-90-R were implemented mainly in clinical settings for the evaluation of clinical effects with repeated implementations before and after treatment, a short version was necessary to prevent a stress overload of the patients and false ratings.

A short form of the Brief Symptom Inventory (BSI) with 53 items was developed by Derogatis and Melisaratos [6] using a factor analysis and maintaining the scale structure with a reduced item number of the SCL-90 (somatization, obsessive-compulsive, interpersonal sensitivity, depression, anxiety, anger-hostility, phobic anxiety paranoid ideation, and psychoticism). In Germany, the BSI is predominantly used for quality management in psychotherapy (e.g. [7]) or other health interventions such as transplantations, chemotherapy, etc. [8–11].

In order to reduce and prevent a stress overload of the patients, and to ensure an easy screening-tool, the BSI-18 [12–14], which measures psychological distress, was developed with highest clinical relevance. The BSI-18 contains only the three six-item scales somatization (SOMA), anxiety (ANX), and depression (DEPR) as well as the global scale General Symptom Index (GSI). Several studies demonstrated that the BSI-18 is a suitable instrument for measuring psychological distress and comorbidities in patients with different mental and somatic illnesses, e.g., organ transplantations [8, 9], cancer [10, 11], and psychotherapy [15, 16]. The instrument was also used in longitudinal studies (e.g. [17, 18]).

The international psychometric properties of the BSI-18 have been discussed in more than ten publications. However, most of the studies examined a broad age range and did not solely focus on the elderly [10–13, 15, 16, 18–22]. Only Petkus et al. [23] (2010 - *M* = 74.4 ± 8.3 years) focused on their subjects’ age by investigating homebound elderly people, however, without examining the psychometric properties and norm values of this specific age group.

In individuals from 18 to 60 years of age the reliability (Cronbach’s α) ranged between the different scales α_*min*_ = .61 [22] and α_*max*_ = .94 [24]. Since the reliability in most of the scales ranged above .80, the reliability can be evaluated as good. The reliability for the American norm sample (*N* = 1134; α-SOMA = .74, α-DEPR = .84, α-ANX = .79, α -GSI = .89; [13]) must be rated as satisfactory. The retest-reliability in psychologically distressed patients after 15 days without intervention (*n* = 102) was satisfactory with values between *r*_*tt*_ = .68 and *r*_*tt*_ = .82 [17].

For factor validity, a strong first factor was discussed (e.g.[25]) – similar to the SCL-90, SCL-90-R, and the BSI-53 [24]. The original three-scale structure was replicated in hospitalized psychosomatic patients (*n* = 638; [15]), and the original scale structure was tested as well by a confirmatory factor analysis (CFA) [8, 26]. The convergent validity of test scores could be shown by several studies [17, 21, 27] likewise sensitivity and specificity [11]. The one existing investigation focusing on elderly people [23] examined only a small sample and ought to be consolidated by further studies.

Considering that populations are growing older and the specific psychological disorders this entails [28], a valid assessment of psychological distress and depressive symptoms is crucial. In the German representative population ranging in age from 50 to 92, between 10 and 16% were classified as depressed ([28] - *M* = 64.4 ± 9.2 years). In order to specify clinically relevant depression, the BSI-18 and the structured clinical interview for Diagnostic and Statistical Manual of Mental Disorders-IV (DSM-IV) (SCID; [29]) were implemented in a small sample of homebound elderly individuals (*n* = 142; [23]). The factor analysis confirmed the three-scale system (depression, anxiety, and somatization) of the BSI-18 (S-B χ^2^ = 136.17; *p* = 0.36). Specifically, the depression and anxiety subscales showed high internal consistency (α = 0.69). Furthermore, the DSM-IV based diagnoses could be predicted in receiver operator curve (ROC) analyses by the BSI depression and anxiety scale (Depression *AUC*¼ 0.89 (area under the curve), *p* < .001; Anxiety *AUC*¼ 0.80, *p* < .001).

Besides age, an affiliation with a lower social class plays a crucial role in the prevalence of psychological distress [30]. The age-specific social inequalities and the physical as well as mental health status were examined by using a two-factor analysis of covariance (*N* = 2222; [31]). Next to the decrease in the physiological and mental health status at a higher age, significant differences between the social classes determined a higher occurrence of health problems in the lower social classes [30].

A meta-analysis [32] based on 300 empirical studies evaluated subjective well-being (SWB) in respect to gender. Although other instruments than the BSI-18 (e.g. the Life-Satisfaction Index or Rosenberg’s Self-Esteem Scale) were used – elderly women showed significantly lower subjective well-being than elderly men [30].

Even though the BSI-18 is internationally one of the most implemented instruments for the assessment of psychological distress, norm values and psychometric properties of a representative sample at older age respective gender and social class have not yet been published. Based on Petkus et al. [23], we are proposing good item and scale characteristics along with a factor structure for the BSI-18 (H1). Since clear specificities for gender are described [30], a measurement invariance across age and gender groups must be present (H2). A further aim of this study was to test the relationship between sociodemographic variables (specifically people ranging between 60 and 95 in age) and psychological distress (H3). Based on Schmidt et al. [31], we expected that older respondents and also those with a lower socio-economic status would report more distress (H3).

## Methods

### Data acquisition

A representative sample of the general population of Germany was collected in November/ December 2009 by a demography consulting company (USUMA, Berlin). Per random-route procedure, households and members of the households were selected. The sample was representative for the German community regarding age, gender, and education, proved by comparisons with the Federal Statistical Office. Firstly, 4091 addresses were selected and the participants were first contacted via mail; 22% dropped out neutral (e.g. person unknown) and 38% could not be asked (e.g. illness, vacation, refusal, unavailability). The participants had to send back general information via mail. The data collection followed in a face to face interview. Finally, a total of 2516 participants were interviewed, whereby 884 individuals could be included in this examination, focusing on the age range between 60 and 95. Since this sample shows a representability for age in the general German population, the percentage of individuals at an older age are representative for the German population as well. In all subsamples of age, the sociodemographic characteristics of this age are representative for the German population.

## Materials

### Sample description

The sample contains 884 individuals (55% female, mean age 71.2, *SD* = 7.4; 45% male, 70.1 average age, *SD* = 6.6) with an overall mean age of 70.8 (*SD* = 7.1, age range = 60–95). Five age groups were established aged: 60–64, 65–69, 70–74, 75–79, and > 80 57% were married, 3% single, 8% divorced, and 33% separated, and 43% lived by themselves. Work status: 47% were employed, 44% were workers and 9% civil servants. The majority earned 1250–2500 € a month (60%), 28% earned less than 1250 €, and 12% earned more than 2500 €. 48% of the participants were classified as members of the lower class, 38% of the middle class, and 14% of the upper class. Detailed information is available in Table 1.

**Table 1:** Sociodemographic description of the study population

	Male *N* = 402	Female *N* = 482	Sum *N* = 884	Statistical testing
Age	70.19 ± 6.62	71.22 ± 7.41	70.75 ± 7.08	*t* = − 2.17 *p* < .03
			Range = 60–95	
Age Groups				χ^2^ = 19.1 *p* < .001
60–64	84 (21%)	108 (22%)	192 (22%)	
65–69	113 (28%)	108 (22%)	221 (25%)	
70–74	116 (29%)	103 (21%)	219 (25%)	
75–79	44 (11%)	89 (19%)	133 (15%)	
≥ 80	45 (11%)	74 (15%)	119 (14%)	
Marital status				χ^2^ = 91.2 *p* < .0001
Single	13 (3%)	10 (2%)	23 (3%)	
Married	292 (73%)	208 (44%)	500 (57%)	
Divorced	27 (7%)	40 (8%)	67 (8%)	
Widowed	70 (17%)	224 (47%)	294 (34%)	
Living				χ ^2^ = 84.84 *p* < .0001
With Partner	298 (74%)	209 (43%)	507 (57%)	
Alone	104 (26%)	273 (57%)	377 (43%)	
Years of schooling				χ ^2^ = 30.95 *p* < .0001
8	247 (61%)	364 (76%)	611 (70%)	
10	98 (24%)	96 (20%)	194 (22%)	
13	57 (14%)	22 (5%)	79 (9%)	
Work Status				χ ^2^ = 109.36 *p* < .0001
Worker	221 (55%)	165 (34%)	386 (44%)	
Employee	117 (29%)	300 (62%)	417 (47%)	
Civil	64 (16%)	17 (4%)	81 (9%)	
Servant/Freelancer				
Income				χ^2^ = 47.8 *p* < .0001
< 1250 €/month	63 (17%)	178 (38%)	241 (28%)	
1250–2500 €/month	266 (70%)	244 (52%)	510 (60%)	
> 2500 €/month	52 (14%)	46 (10%)	98 (12%)	
Class index				χ ^2^ = 31.79 *p* < .0001
Lower class	196 (49%)	231 (48%)	427 (48%)	
Middle class	125 (31%)	211 (44%)	336 (38%)	
Upper class	81 (20%)	40 (8%)	121 (14%)	

### Psychological assessments

The demographic information and the BSI-18 were collected by the survey. The BSI-18 [14] consists of three six-item scales: somatization, anxiety, and depression. All the questions apply to the two preceding weeks and were to be rated by using “0 = not at all”, “1 = several days”, “2 = more than half the days”, and “3 = nearly every day”. The BSI-18 somatization, anxiety, and depression scores and the global Scale General Symptom Index (GSI) were calculated by sum scores. The GSI therefore ranges between 0 and 72 and the three scales between 0 and 24. Concerning the good validity of the BSI, evidence had been based on external criteria such as the Patient Health Questionnaire (PHQ-4; [33, 34]). Internal consistency was α = .82 for somatization, α = .87 for depression, α = .84 for anxiety and α = .93 for the GSI.

### Statistics

Most of the statistical calculations were conducted using IBM SPSS Statistics 20. Only the confirmatory factor analysis (CFA) was performed with R using the lavaan package [35].

First, a Missing Data Analysis led to the exclusion of four participants as they showed more than the tolerated amount of missing data (tolerated < 2 items of each scale, < 6 items in total). At last, a total of 0.09% of the answers were missing and not assigned randomly (Little MCAR-Test: χ^2^ = 550.971, *df* = 333, *p* < .0001). Therefore, they were replaced by using Multiple Imputation to avoid selection bias (MCMC in LISREL 8.15; [36]). Descriptive statistics and reliability of the test scores were calculated. Construct validity was tested by using exploratory and confirmatory factor analysis.

Due to the lack of multivariate normality, robust maximum likelihood estimation (MLR; [37]) was used for model testing. According to Schermelleh-Engel, Moosbrugger, and Müller [38], a good (acceptable) model fit is achieved with χ^2^/df index below 2.0 (below 3.0), Comparative Fit Index (CFI) as well as Tucker-Lewis-Index (TLI) above .95 (above .90), Standardized Root Mean Square Residual (SRMR) below .05 (below .10), and Root Mean Square Error of Approximation (RMSEA) below .05 (below .08).

Second, for the analysis of measurement invariance across age and gender groups, we followed the recommended procedures [39]. The age intervals were chosen in such a way as to make them as fine-grained as possible, while maintaining comparable interval sizes (in terms of years included) and respectable sample sizes. Namely, we compared four increasingly restrictive models to test for configural invariance (without restrictions), metric invariance (equal loadings), scalar invariance (equal loadings and intercepts), and strict invariance (equal loadings, intercepts, and residual variances). Comparisons were judged using differences in χ^2^, CFI, and gamma hat (GH; [40]). χ^2^ should not differ significantly when adding one additional restriction, and differences in CFI as well as in GH should not exceed .01. All correlations are reported using the Pearson product-moment correlation coefficient. Tests of significance use an α level of .05.

Third, the sociodemographic parameters such as age, gender, and socioeconomic status [31] were tested via t-test or single-factor variance analysis. The age-dependent differences led to age-specific standards.

## Results

Item- and scale characteristics and exploratory factor structure (H1).

The item- and scale statistics, the reliability of test scores, and the results of the exploratory three-factorial factor analysis are shown in Table 2. Herein, the mean values and standard deviations of the three scales are listed in the third column. Item no. 16: “Feeling weak in parts of your body” reached the maximum with 0.57 ± 0.82 whereas item no. 1: “Faintness or dizziness” represented the minimum with 0.31 ± 0.62 (scale 1 “Somatization” in total: *∑ 2.36* ± 3.07 and a **ω** -value of .826). The maximum mean value of the second scale (“Depression”) was measured by item no. 5: “Feeling lonely” (0.55 ± 0.92) whereas the minimum was in item no. 17: “Thoughts of ending your life” (0.08 ± 0.34). This scale reached a ω-value of .890 and a total sum of ∑2.04 ± 3.34.

**Table 2:** Item- and scale statistics, reliability and results of the exploratory three-factorial factor analysis in the total sample (*N* = 884)

no item	*M* (*SD*)	corrected item-total correlation	ω without the item	exploratory factor analysis			
Scale 1: Somatization (ω = .826)	*∑2.36* ± 3.07			*h* ^*2*^	*f1*	*f2*	*f3*
1 Faintness or dizziness	0.31 ± 0.62	.59	.798	.57		.70	
4 Pains in heart or chest	0.41 ± 0.70	.62	.794	.63		.77	
7 Nausea or upset stomach	0.34 ± 0.66	.52	.811	.45		.52	
10 Trouble getting your breath	0.39 ± 0.72	.64	.788	.63		.77	
13 Numbness or tingling in parts of your body	0.34 ± 0.69	.50	.815	.42		.54	
16 Feeling weak in parts of your body	0.57 ± 0.82	.66	.781	.61		.67	
Scale 2: Depression (ω = .890)	*∑2.04* ± 3.34						
2 Feeling no interest in things	0.42 ± 0.73	.72	.868	.69	.73		
5 Feeling lonely	0.55 ± 0.92	.68	.881	.70	.81		
8 Feeling blue	0.34 ± 0.72	.78	.857	.72	.73		
11 Feelings of worthlessness	0.27 ± 0.65	.74	.866	.68	.68		
14 Feeling hopeless about the future	0.38 ± 0.79	.76	.862	.68	.72		
17 Thoughts of ending your life	0.08 ± 0.34	.43	*.*887	.58			.74
Scale 3: Anxiety (ω = .857)	*∑1.55* ± 2.72						
3 Nervousness or shakiness inside	0.31 ± 0.62	.63	.838	.58		.54	
6 Feeling tense or keyed up	0.45 ± 0.74	.58	.855	.50	.59		
9 Suddenly scared for no reason	0.23 ± 0.57	.69	.828	.68			.72
12 Spells of terror or panic	0.13 ± 0.43	.71	.831	.68			.71
15 Feeling so restless you could not sit still	0.23 ± 0.61	.71	.820	.62			.67
18 Feeling fearful	0.21 ± 0.55	.64	.833	.66			.72
Global Severity Index (ω = .929)	*∑5.96* ± 8.08						

*Note:.* Only factor loadings *> .40* are reported

The third scale (“Anxiety”) reached the maximum mean value in item no. 6: “Feeling tense or keyed up” (0.45 ± 0.74) and its minimum in item no. 12: “Spells of terror or panic” (0.13 ± 0.43). The total mean value sum and its standard deviation for the third scale is ∑1.55 ± 2.72, the **ω**-value .857. The Global Severity Index reached a sum mean value of ∑5.96 ± 8.08 and an **ω** of .929.

Looking at the exploratory factor analysis, it is noteworthy that all the items of the Somatization scale are allocated to *f*2 while the Depression scale is assigned to *f*1. Only the item “Thoughts of ending your life” is allocated to *f*3. In the anxiety scale the items “Nervousness or shakiness inside” (*f*2) and “Feeling tense or keyed up” (*f*1) were differently assigned than the rest of the scale (*f*3).

All the items of all three subscales evidenced good item-total correlations in excess of .50 – with the exception of item 17, which still had an acceptable value. In no case did the deletion of an item lead to a higher reliability coefficient than what was found for the full subscale (Table 2).

### Factorial structure (H1)

While the cross-loadings discovered in EFA represent some cause for concern, we elected to test models that conform to the mapping of items onto their respectively theorized components. Findings by Franke and colleagues [41] support this notion.

Table 3 shows the results of CFA regarding the scale structure and the age-depending factor structure of the BSI-18. The only models that should be considered acceptable among the ones tested are the three-factor-models (SOMA/DEPR/ANX) with correlated factors and a general GSI factor. This model fulfills the criteria for acceptability for all the fit indices employed: RMSEA and SRMR are good, CFI and TLI are acceptable, and CMIN/DF is barely acceptable. The χ^2^-statistic is highly significant, which is not surprising given the large sample size [42]. Accordingly, we put more emphasis on the fit indices, which showacceptable, even good, fit overall. Thus, we were able to confirm the theoretical model of the BSI-18 in a sample of elderly people. Factor loadings of the indicator variables ranged between .52 and 77. The GSI factor correlated highly with the three subscales, *r*_SOMA_ = .77, *r*_DEPR_ = .92, *r*_ANX_ = .97. Intercorrelations between the subscales ranged between .70 and .88.

**Table 3:** Results of CFA regarding scale structure of the BSI-18 (*N* = 884)

Model	χ^2^(*df*)	CMIN/DF	CFI	TLI	RMSEA [90% CI]	SRMR
1-Factor-Model	664.77 (135) *	4.78	.838	.817	.067 [.063; .070]	.067
2-Factor-Model (DEPR + ANX/ SOMA)	471.21 (134) *	3.52	.897	.883	.053 [.050; .057]	.054
3-Factor-Model (SOMA/ DEPR/ ANX)	401.86 (132) *	3.04	.918	.905	.048 [.045; .052]	.053
GSI-Factor 1. Order und 2-Factor-Model 2. Order (DEPR + ANX/ SOMA)	467.69 (133) *	3.52	.898	.883	.053 [.050; .057]	.054
GSI-Factor 1. Order und 3-Factor-Model 2. Order (SOMA/ DEPR/ ANX)	401.86 (132) *	3.04	.918	.905	.048 [.045; .052]	.053

*Note*. χ^2^ is Yuan-Bentler-scaled; * = *p* < .001, *CFI* Comparative Fit Index, *TLI* Tucker Lewis Index, *RMSEA* Root Mean Square Error of Approximation, *SRMR* Standarized Root Mean Square Residual

### Factorial invariance (H2)

The results of the test for measurement invariance can be found in Table 4. Some of the configural models were not acceptable when considering the *CFI*. The *GH,* on the other hand, presented evidence for acceptable fit in all groups. We find evidence for strict measurement invariance for the age ranges (60–64, 65–69, 70–74 vs. 75, or older) and gender (female vs. male).

**Table 4:** Analysis of measurement invariance of the BSI-18 three-factorial model between groups of age and gender

Model	χ^2^(*df*)	Δχ^2^	*p*	CFI	ΔCFI	GH	ΔGH
Gender Multi-group Analysis (Female/Male)							
Configural invariance	628.82 (264)			.900		.916	
Female	295.711 (132)			.916		.963	
Male	335.521 (132)			.881		.945	
Metric invariance	647.59 (281)	18.77	.342	.899	.001	.917	.001
Scalar invariance	689.77 (295)	42.18	< .001	.891	.008	.910	.007
Strict invariance	711.41 (313)	21.64	.248	.890	.001	.908	.002
Age Multi-group Analysis (60–64, 65–69, 70–74, ≤ 75)							
Configural invariance	1069.61 (528)			.870		.936	
60–64	291.785 (132)			.916		.953	
65–69	285.490 (132)			.856		.933	
70–74	276.288 (132)			.830		.931	
≤ 75	216.042 (132)			.872		.931	
Metric invariance	1123.38 (579)	53.77	.369	.869	.001	.936	.000
Scalar invariance	1208.76 (621)	85.38	< .001	.859	.010	.931	.005
Strict invariance	1268.30 (675)	59.54	.281	.857	.002	.931	.000

*CFI* Comparative Fit Index, *GH* Gamma hat

### Differences in BSI-18 based on socio-demographic variables (H3)

In the scales somatization (SOMA), depression (DEPR), and the General Symptom Index (GSI) increasing values are shown with increasing age. In the group of 65 to 69 years of age, the values of the scale decrease. In the group of 75 to 79 years of age, the maximum for the depression scale (DEPR *M* = 2.70, *SD* = 3.12) is reached. However, for somatization (*M* = 3.89, *SD* = 3.75) and the General Symptom Index (*M* = 8.14, *SD* = 9.38), the maximum is reached at the age of 80, and older. For the two scales SOMA (*p* < .0001) and DEPR (*p* < .02), a significant increase with increasing age can be observed. In addition, the scale DEPR (*p* < .02) significantly increases until 75 to 79 years of age and decreases again afterwards (see Table 5). In contrast, the anxiety (ANX) scale did not differ significantly between age groups (*p* < .29). In the group of 80 year-olds and older the maximum was reached for the ANX scale (*M* = 1.82, *SD* = 3.14) (see Table 5).

**Table 5:** Age-, gender- and social-class-related BSI-18 differences

BSI-18 Scales	Somatization	Depression	Anxiety	General Symptom Index
Age-related differences				
60–64 (*N* = 192)	1.97 ± 2.78	1.98 ± 3.12	1.74 ± 2.96	5.70 ± 8.01
65–69 (*N* = 221)	1.89 ± 2.65	1.56 ± 2.90	1.40 ± 2.47	4.85 ± 7.15
70–74 (*N* = 219)	2.25 ± 2.98	1.98 ± 3.25	1.31 ± 2.36	5.54 ± 7.71
75–79 (*N* = 133)	2.53 ± 3.15	2.70 ± 3.86	1.71 ± 2.92	6.93 ± 8.65
≥ 80 (*N* = 119)	3.89 ± 3.75	2.43 ± 3.84	1.82 ± 3.14	8.14 ± 9.38
*F*, *df*, *p*-value, effect size	*F* = 10.03, *p* < .0001, η^2^ = .04	*F* = 2.89, *p* < .02, η^2^ = .01	*F* = 1.24, *p* < .29	*F* = 3.94, *p* < .004, η^2^ = .02
Gender-related differences				
Male (*N* = 402)	2.34 ± 2.99	1.78 ± 3.22	1.42 ± 2.72	5.54 ± 7.90
Female (*N* = 482)	2.38 ± 3.13	2.27 ± 3.42	1.66 ± 2.73	6.31 ± 8.23
Sum (*N* = 884)	2.36 ± 3.07	2.04 ± 3.34	1.55 ± 2.72	5.96 ± 8.08
*t*, *p*-value	*t* = −0.20	*t* = −2.18, *p* < .03	*t* = −1.29, p <,20	*t* = −1.41, *p* < .16
Social-class-related differences				
Lower class (*N* = 427)	2.80 ± 3.38	2.52 ± 3.73	1.83 ± 3.10	7.15 ± 9.06
Middle class (*N* = 336)	2.04 ± 2.76	1.69 ± 3.03	1.37 ± 2.46	5.09 ± 7.34
Upper class (*N* = 121)	1.72 ± 2.46	1.36 ± 2.35	1.11 ± 1.71	4.18 ± 5.35
*F*, *p*-value, effect size	*F* = 9.15, *p* < .0001, η^2^ = .02	*F* = 8.98, *p* < .0001, η^2^ = .02	*F* = 4.64, *p* < .01, η^2^ = .01	*F* = 9.71, *p* < .0001, η^2^ = .02

Concerning gender, women showed higher values in the three scales DEPR, ANX, and GSI than men. However, the gender difference reached the level of significance only for the scale DEPR (*p* < .03). Cohen’s *d* was small for these comparisons, *d*s ≤ 0.15. In respect to social class, the values of all four scales significantly increased the lower the social class was. The higher the social class, the fewer symptoms were present in all four scales. The corresponding effect sizes were small, η^2^_*p*_ ≤ .02 (see Table 5).

Based on these findings, norm values specific to individuals between the ages of 60 and 95 were calculated and are shown in Table 6. The table presents T values for all possible BSI scores.

**Table 6:** Normative T-values of the BSI-18.

BSI-18 Somatization scale						
	1	2	3	4	5	0
Age	60–64	65–69	70–74	75–79	80–95	60–95
*N=*	192	221	219	133	119	884
0	42	43	41	41	39	42
1	51	51	49	48	45	49
2	54	54	53	51	48	53
3	57	57	56	54	50	55
4	59	59	58	57	52	57
5	61	61	60	59	53	59
6	62	64	61	62	55	61
7	64	66	64	63	58	63
8	67	67	66	64	60	65
9	69	70	68	65	63	67
10	69	71	69	67	65	69
11	70	74	70	69	66	70
12	73	76	72	70	69	72
13	75	76	74	72	72	74
14	78	76	74	77	74	75
15	80	78	75	80	74	77
16	80	80	78	80	74	79
17	80	80	80	80	76	80
≥18	80	80	80	80	80	80
BSI-18 Somatization scale						
	1	2	3	4	5	0
Age	60–64	65–69	70–74	75–79	80–95	60–95
*N=*	192	221	219	133	119	884
0	43	44	43	42	41	43
1	52	54	52	50	51	52
2	55	57	55	53	54	55
3	57	59	58	56	56	57
4	59	61	59	57	58	59
5	61	63	61	58	61	61
6	62	65	62	59	62	62
7	63	66	64	60	63	63
8	65	67	65	61	64	64
9	67	68	66	62	64	66
10	69	69	68	65	65	67
11	70	70	69	67	65	68
12	71	70	69	68	66	69
13	71	71	71	70	67	70
14	73	72	74	72	68	72
15	75	74	74	73	69	73
16	78	78	74	74	71	75
17	80	80	74	74	72	76
18	80	80	76	74	76	78
19	80	80	80	77	80	80
≥20	80	80	80	80	80	80
BSI-18 Anxiety sub-scale						
	1	2	3	4	5	0
Age	60–64	65–69	70–74	75–79	80–95	60–95
*N=*	192	221	219	133	119	884
0	43	44	44	43	43	44
1	52	53	54	52	52	53
2	56	57	58	57	57	57
3	59	60	61	59	59	60
4	61	62	62	61	60	61
5	62	64	64	61	62	62
6	63	66	64	63	64	64
7	65	67	66	65	65	66
8	67	67	68	65	65	66
9	68	68	70	66	66	68
10	69	70	75	68	67	69
11	70	71	76	69	68	71
12	70	74	76	71	70	72
13	71	78	76	73	71	74
14	74	80	78	77	72	76
15	75	80	80	80	74	78
16	75	80	80	80	74	78
17	75	80	80	80	76	79
18	78	80	80	80	80	80
≥19	80	80	80	80	80	80
BSI-18 General Symptom Index scale						
	1	2	3	4	5	0
Age	60–64	65–69	70–74	75–79	80–95	60–95
*N=*	192	221	219	133	119	884
0	39	41	39	39	37	39
1	46	47	45	44	43	46
2	49	50	48	47	45	48
3	51	52	51	49	47	50
4	52	53	53	50	49	52
5	53	55	54	53	49	53
6	55	56	56	54	50	55
7	56	58	57	54	51	56
8	58	58	58	55	53	57
9	58	59	58	56	54	58
10	59	60	59	57	55	58
11	60	61	60	58	56	59
12	60	62	61	59	57	60
13	61	62	61	59	58	61
14	61	63	62	60	59	61
15	62	64	62	60	60	62
16	62	64	62	61	60	62
17	63	64	63	61	61	63
18	64	65	63	61	62	63
19	64	66	64	62	62	64
20	65	66	64	62	62	64
21	66	66	65	62	63	65
22	66	66	65	63	63	65
23	66	67	66	64	63	66
24	67	67	67	64	64	66
25	67	68	67	64	65	66
26	67	68	67	65	66	67
27	68	69	68	65	66	67
28	69	69	69	65	66	68
29	70	70	71	67	66	69
30	70	70	72	69	67	70
31	70	71	75	71	67	71
32	70	71	76	73	67	71
33	71	73	76	74	67	72
34	71	78	76	77	67	73
35	71	80	76	80	68	73
36	72	80	76	80	69	74
37	74	80	76	80	69	75
38	75	80	76	80	71	76
39	75	80	76	80	74	77
40	75	80	76	80	74	77
41	75	80	76	80	74	77
42	75	80	76	80	74	77
43	75	80	76	80	74	77
44	75	80	76	80	74	77
45	75	80	76	80	76	78
46	78	80	76	80	80	79
47	80	80	76	80	80	80
48	80	80	78	80	80	80
≥49	80	80	80	80	80	80

*Note. T* value distribution (*M* = 50, *SD* = 10) for the BSI-18 scales

## Discussion

We assessed the Brief Symptom Inventory-18 as a measure regarding psychological distress for elderly individuals. This is the first study investigating a representative sample at an age range of 60 to 95. Two earlier studies with samples at a younger age range examined the psychometric properties and benefits of the instrument [8, 16]. However, the psychometric properties, norm values, and factorial structure of individuals between the ages of 60 and 95 have never been reported so far.

The present study aims to address this lacuna by examining a sample of older individuals. First based on Petkus et al. [23], we proposed good item and scale characteristics, along with factor structure for the BSI-18 (H1). The item and scale properties of the BSI-18 were found to be satisfactory, as was the reliability of test scores as assessed by McDonalds’s ω. We tested several potential models and found that a three-factor-model would suit the data best. Therefore for best fit, the model of three factors loading on a general GSI factor was chosen.

Since clear specificities for gender are described [30], measurement invariance across age and gender groups must be present (H2). The present results confirm strict measurement invariance for this model across gender and age, allowing for comparisons between the groups. When considering the CFI, the configural model evinced unacceptable fit for some groups. The GH index, on the other hand, presented evidence for acceptable, even good fit, in all groups. The results can, thus, not be considered entirely unambiguous, and researchers should keep this in mind, when comparing the BSI-18 between groups.

Based on Schmidt et al. [31], we expected that older respondents and those with a lower socio-economic status would report more distress (H3). In the present cross-sectional study, the individuals from 65 to 69 years of age showed significantly higher values for somatization, depression, and the General Symptom Index compared to individuals aged 60–64. Subsequently, the individuals of 70–74 years of age also showed significant higher values for somatization, depression, and the General Symptom Index compared to individuals at 60–64 years of age. This fits to the well-described link between psychological distress and physical symptoms or somatoform disorders [43–48]. The age group of 75–79 years of age reported the highest values for depression. In this age group, social contacts are diminishing, and concerns about needing care in old age may be an influencing factor. Although the highest values of somatization and the General Symptom Index were reported at the age of 80, and older, for this age range, the depression values are lower compared to individuals 75–79 years of age (Table 3). Possibly, people of this age have resigned themselves to life and common daily problems so that there is less psychological distress. In contrast, anxiety symptoms did not differ significantly between the age groups.

Considering the results of the gender-related BSI-18 differences (Table 3), women consistently have higher values, with a statistical significant variation in the Depression-scale (*p* < 0.03). This confirms the results by Pinquart and Sörensen [32] who analyzed significantly lower subjective well-being and a less positive self-concept in women than in men. A possible reason may be the loss of a male life partner: in our subjects, the percentage of widowed women was more than double that of the male group (47% widows and 17% widowers). Glaesmer and colleagues [28] (*M* = 64.4 ± 9.2 years, age range: 50–92) found a link between psychological distress and elderly people living by themselves. Even in the present sample, more women than men lived alone (57% women to 26% men). Although a partnership may have a potentially protective effect on psychological distress [28], the effect size measured in this context, however, is regarded as low.

In respect to social class, the higher the social class, the fewer symptoms are present in all four scales. These are in line with the results by Schmidt and colleagues [31]. There may be more health and financial problems connected to the lower social class associated with elevated psychological distress [30, 49].

Even though it is a representative German sample, it has the limit that neither a physician nor an fMRI evaluated possible cognitive impairment or mild dementia.

## Conclusion

The item and scale properties as well as the reliability of the test scores of the BSI-18 were found to be satisfactory. The three-factor-model fits the data the best. The measurement invariance for this model across gender and age was confirmed, allowing comparisons between these groups. Individuals older than 60 years of age, women, and individuals from a lower social class showed significantly increasing values for somatization, depression, and the General Symptom Index.

Thus, the findings underline the need for preventive mechanisms for elderly people such as (re)activating their social networks or strengthening their physical and psychological well-being, for example, by involving them in physical group activities [50, 51].

## Abbreviations

ANX: Anxiety
AUC: Area under the curve
BSI: Brief Symptom Inventory
CFA: Confirmatory factor analysis
CFI: Comparative Fit Index
CMIN/DF: Minimum discrepancy divided by its degrees of freedom
DEPR: Depression
DSM: Diagnostic and Statistical Manual of Mental Disorders
GH: Gamma hat
GSI: General Symptom Index
LISREL: Linear structural relations, statistical software package
MCAR: Missing completely at random
MCMC: Markov chain Monte Carlo
MLR: Maximum likelihood estimation
PHQ: Patient Health Questionnaire
RMSEA: Root Mean Square Error of Approximation
ROC: Receiver operator curve
SCID: Structured clinical interview
SCL: Symptom checklist
SOMA: Somatization
SRMR: Standardized Root Mean Square Residual
SWB: Subjective well-being
TLI: Tucker Lewis Index
USUMA: USUMA Sozial- und Marktforschungsinstitut
